# Potential of Large Language Models in Generating Multiple-Choice Questions for the Japanese National Licensure Examination for Physical Therapists

**DOI:** 10.7759/cureus.79183

**Published:** 2025-02-17

**Authors:** Shogo Sawamura, Kengo Kohiyama, Takahiro Takenaka, Tatsuya Sera, Tadatoshi Inoue, Takashi Nagai

**Affiliations:** 1 Department of Rehabilitation, Heisei College of Health Sciences, Gifu, JPN

**Keywords:** chatgpt, custom gpts, japanese national licensure examination for physical therapists, large language models (llms), multiple-choice questions

## Abstract

Introduction

This study explored the potential of using large language models (LLMs) to generate multiple-choice questions (MCQs) for the Japanese National Licensure Examination for Physical Therapists. Specifically, it evaluated the performance of a customized ChatGPT (OpenAI, San Francisco, CA, USA) model named "Physio Exam Generative Pre-trained Transformers (GPT)" in generating high-quality MCQs in non-English contexts.

Materials and methods

Based on the data extracted from the 57th and 58th Japanese National Licensure Examination for Physical Therapists, 340 MCQs, including correct answers, explanations, and associated topics, were incorporated into the knowledge base of the GPTs. The prompts and outputs were conducted in Japanese. The generated MCQs covered major topics in general (anatomy, physiology, and kinesiology) and practical questions (musculoskeletal disorders, central nervous system disorders, and internal organ disorders). The quality of the MCQs and their explanations were evaluated by two independent reviewers using a 10-point Likert scale across five criteria: clarity, relevance to clinical practice, suitability of difficulty, quality of distractors, and adequacy of rationale.

Results

The generated MCQs achieved 100% accuracy for both general and practical questions. The average scores across the evaluation criteria ranged from 7.0 to 9.8 for general questions and 6.7 to 9.8 for practical questions. Although some areas exhibited lower scores, the overall results were favorable.

Conclusions

This study demonstrates the potential of LLMs to efficiently generate high-quality MCQs, even in non-English environments such as Japanese. These findings suggest that LLMs can adapt to diverse linguistic settings, reduce educators' workload, and improve the quality of educational resources. These results lay a foundation for expanding the application of LLMs to educational settings across non-English-speaking regions.

## Introduction

Multiple-choice questions (MCQs) are widely regarded as key tools for objectively assessing student knowledge and comprehension in medical education [1]. As such, they are extensively used in national licensure examinations and various assessment tests, particularly for healthcare professionals [2], including licensure examinations for physical therapists [3]. However, creating many high-quality MCQs presents a significant challenge for educators, highlighting the need to improve efficiency in educational settings.

In recent years, large language models (LLMs), such as ChatGPT (OpenAI, San Francisco, CA, USA), have demonstrated high performance in healthcare-related examinations, with capabilities sufficient to pass various tests, including the national licensure examination for physical therapists [3,4]. Leveraging such technologies holds significant potential for streamlining the generation of MCQs and alleviating educators’ workloads [5,6]. By reducing the time required for question creation, educators can dedicate more time to student guidance and research, ultimately helping to improve educational quality and broader societal benefits [7,8]. However, previous studies have highlighted the limitations in the accuracy and quality of the generated questions, indicating that they have not yet reached a practical level for widespread use [5,9-11]. One potential solution for these challenges is the retrieval-augmented generation (RAG). The RAG integrates external knowledge into the model's response generation process, enhancing the generated content's accuracy and consistency [12]. Furthermore, it has been reported that refining prompt design can significantly enhance the performance of LLMs [13,14]. However, prior studies have predominantly centered around languages with linguistic structures similar to English, with limited applications in structurally distinct languages like Japanese.

In this study, we explored the potential of LLMs in linguistic environments distinct from English by developing a customized version of ChatGPT (hereafter referred to as Generative Pre-trained Transformers (GPTs)) based on the Japanese language. GPTs are tailored models of ChatGPT designed for specific tasks [15]. This study configured them to efficiently generate MCQs related to the Japanese National Licensure Examination for Physical Therapists. The GPTs were embedded with the pre-collected MCQ data, correct answers, and explanations, enabling them to generate new questions based on the provided prompts and relevant information.

This approach was adopted to ensure that the generated questions accurately adhered to the format and content of past national licensure examinations. By embedding pre-collected MCQ data, we aimed to enhance the reliability and validity of generated questions, ensuring alignment with actual exam standards. Additionally, using GPTs allows for the easy sharing of resources via links, facilitating practical applications in educational settings.

This study aimed to evaluate the potential application of LLMs by using GPTs to generate MCQs in languages structurally distinct from English. Specifically, MCQs were designed to be similar to those found in the Japanese National Licensure Examination for Physical Therapists, and their accuracy was examined to clarify the potential for application in licensure exam preparation. Thus, this study aimed not only to validate the feasibility of LLMs in such linguistic environments but also to contribute to improving the efficiency of physical therapy licensure exam preparation.

## Materials and methods

Structure and content of the Japanese National Licensure Examination for Physical Therapists

The Japanese National Licensure Examination for Physical Therapists comprises 160 general and 40 practical questions, all presented as MCQs. This format is determined by the Ministry of Health, Labour, and Welfare of Japan, which oversees the licensure of physical therapists. The official examination questions are publicly available on the Ministry of Health, Labour, and Welfare of Japan website.

The general questions cover various topics, including anatomy, physiology, kinesiology, pathology, clinical psychology, rehabilitation medicine, clinical medicine, and physical therapy. These questions assess the fundamental knowledge and comprehension essential for physical therapy practice. Conversely, practical questions focus on clinical applications and require advanced professional expertise. For example, some questions involved brief case descriptions and asked candidates to identify the most appropriate physical therapy intervention. The examination included two types of MCQs: A-type questions, where candidates select one correct answer out of five options, and X2-type questions, which require two correct answers out of five options.

Configuration and structure of GPTs

A customized version of ChatGPT, referred to as GPTs [15], was used in the present study, and the resulting model was named "Physio Exam GPT." The customization process involved two primary components: first, a knowledge base comprising 340 MCQs and corresponding correct answers, explanations, and linked topics derived from the 57th and 58th Japanese National Licensure Examination for Physical Therapists was constructed. The authors developed these explanations and the associated topics, as presented in the Appendices section (Supplementary 1). Second, a tailored prompt configuration was designed to enable users to input relevant topics, allowing the GPTs to generate MCQs based on the information embedded within the knowledge base. The specifics of the prompt design are presented in Supplementary 2; as described, the customization process was intentionally limited to embedding the "knowledge" (MCQs) and configuring the "prompts," with no additional fine-tuning or model adjustments. The generated questions relied solely on the standard functionalities of the custom GPT framework.

Data collection

The general questions section comprised 20 questions and explanations generated for each of the primary domains: “anatomy,” “physiology,” and “kinesiology,” yielding 60 general questions. These domains were selected based on their essential role in understanding human movement and bodily functions, forming the foundation of physical therapy education. Similarly, the practical questions section comprised 10 questions and explanations generated for each of the key domains: “musculoskeletal disorders,” “central nervous system disorders,” and “internal organ disorders,” yielding 30 practical questions.

The data used in this study were collected on January 2, 2025. Unlike other standardized exams, the Japanese National Licensure Examination for Physical Therapists does not explicitly specify question distribution by subject. Instead, questions are generally designed to integrate multiple fields, such as physiology and internal medicine. Given this context, we initially conducted a pilot study focusing on the core foundational subjects of physical therapy (anatomy, physiology, and kinesiology) to assess the feasibility of LLM-generated questions.

Evaluation of questions and explanations

Three independent reviewers evaluated the questions and explanations generated by the Physio Exam GPT. Initially, the reviewers independently assessed the accuracy of the answers to the questions, categorizing them as correct or incorrect. The quality of the questions and explanations was evaluated based on the following criteria derived from previous research, and scores were assigned using a 10-point Likert scale.

The questions and explanations generated by the Physio Exam GPT were evaluated through an assessment conducted by three independent reviewers. The reviewers first independently determined the accuracy of each question, classifying them as correct or incorrect. Subsequently, the quality of the questions and explanations was assessed using five criteria derived from prior research, with scores assigned on a 10-point Likert scale [9,16]. This evaluation aimed to assess whether GPT-generated questions aligned with the quality and structure of actual national licensure exam questions, using the latter as a reference standard. This study did not aim to assess the actual national licensure examination questions using the same scale but to compare the generated questions against them. To further account for the novelty of the generated questions, an additional evaluation criterion, "similarity," was introduced, resulting in a comprehensive evaluation based on the six following criteria:

The first criterion, clarity of the item, examines whether the question stem provides sufficient information for a well-prepared candidate to identify the single best answer. The second, relevance to clinical practice, evaluates the extent to which the item applies to the clinical practice of physical therapists. The third, suitability based on difficulty, assesses whether the item's difficulty level is appropriate for inclusion in the Japanese National Licensure Examination for Physical Therapists. The fourth, quality of the distractors, considers whether the distractors are plausible and consistent in concept and structure. The fifth, adequacy of the rationale, determines whether the explanation effectively justifies why the correct answer is correct and why the distractors are incorrect. Finally, the sixth criterion, similarity, measures how closely the generated question resembles existing items in the knowledge base.

The three reviewers were faculty members at a physical therapy training institution, holding physical therapy licenses and having extensive familiarity with the Japanese National Licensure Examination for Physical Therapists. All reviewers had over 10 years of clinical experience and specialized in different areas of physical therapy (musculoskeletal disorders, central nervous system disorders, and internal organ disorders). Before the evaluation, the reviewers held thorough discussions to align their understanding of the evaluation criteria and ensure assessment consistency.

## Results

**Figure 1:**
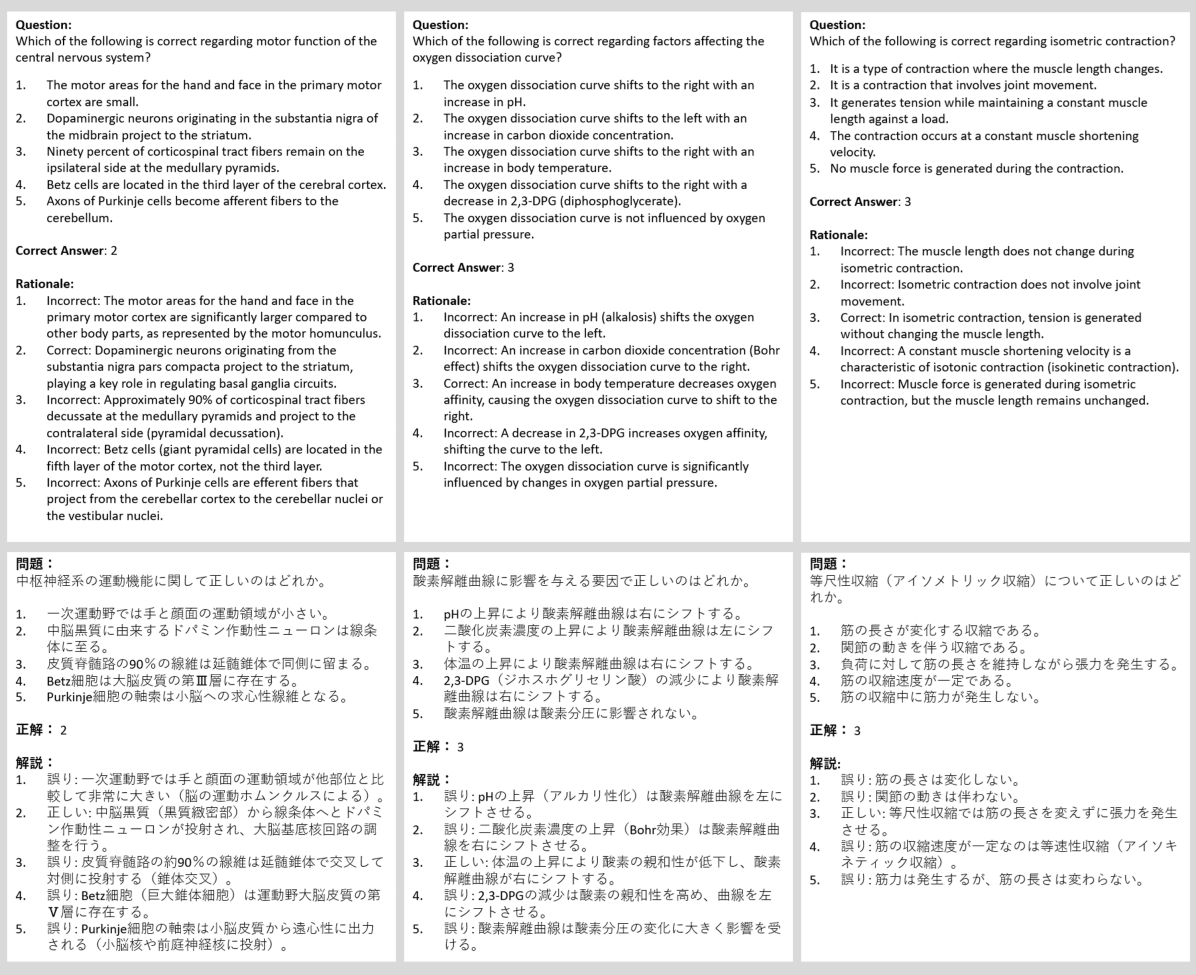
General questions created by Physio Exam GPT The upper section presents the English translation of the AI-generated MCQs, while the lower section displays the original Japanese output. The correct answer and a rationale follow each question. GPT: generative pre-trained transformers, AI: artificial intelligence, MCQs: multiple-choice questions

**Figure 2:**
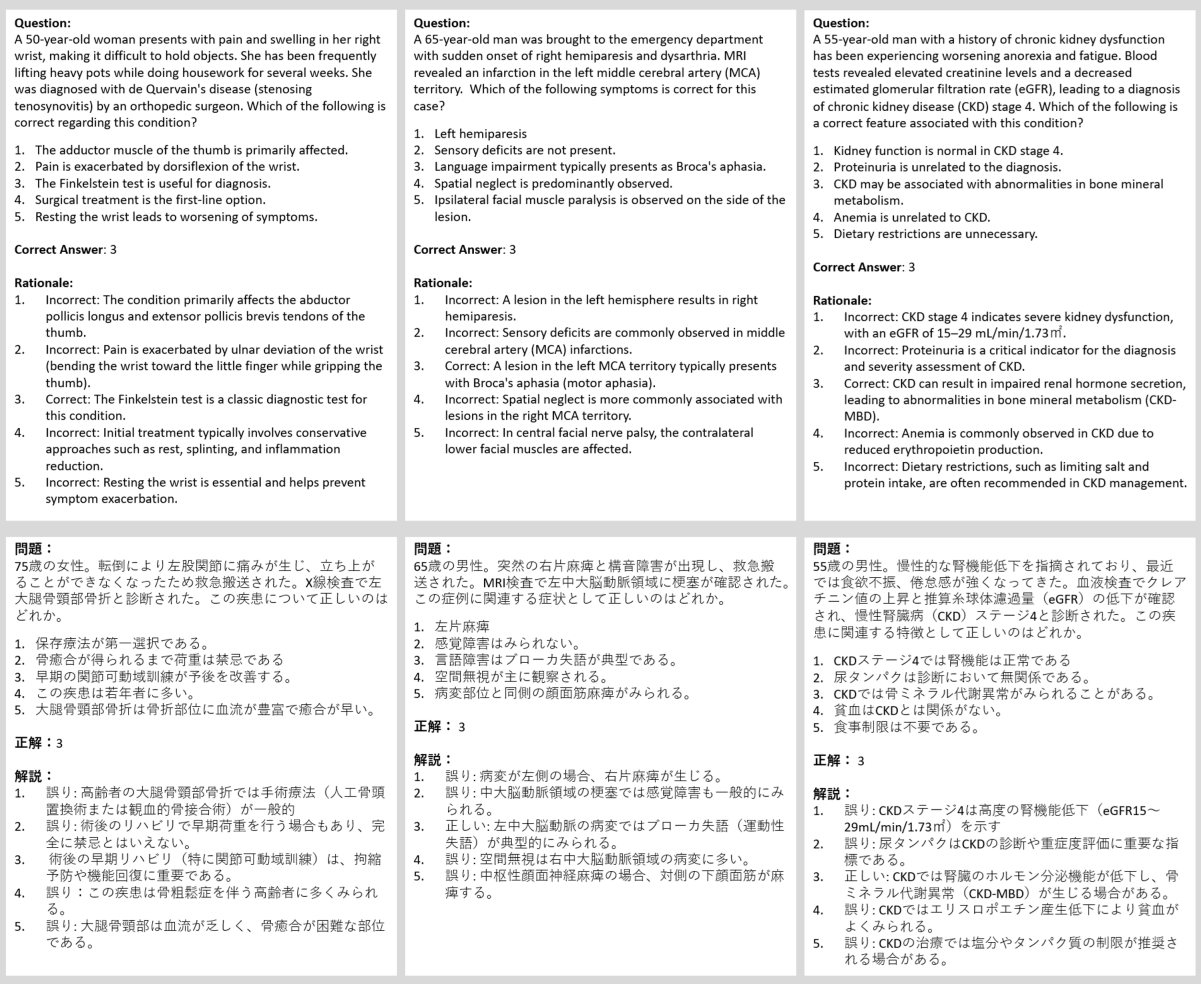
Practical questions created by Physio Exam GPT The upper section presents the English translation of the AI-generated MCQs, while the lower section displays the original Japanese output. The correct answer and a rationale follow each question. GPT: generative pre-trained transformers, AI: artificial intelligence, MCQs: multiple-choice questions

The primary questions generated by the Physio Exam GPT are shown in Figures 1-2. The scoring results of the two reviewers are presented in Tables 1-2. The accuracy of the correct answers for both the general and practical questions, which refers to the correctness of the answers generated by Physio Exam GPT for its own generated questions, was 100.0%.

**Table 1:** Scores for each of the evaluation criteria in general questions Three evaluators assigned scores using a 10-point Likert scale (mean ± standard deviation)

Topics	Accuracy of the answer	Clarity	Relevance	Suitability level	Quality of distractors	Adequacy of rationale	Similarity
Anatomy (n=20)	100.0% (20/20)	9.0 ± 1.2	8.5 ± 1.5	8.7 ± 1.1	8.4 ± 1.5	8.3 ± 1.2	4.0 ± 1.8
Physiology (n=20)	100.0% (20/20)	8.7 ± 1.4	8.5 ± 1.2	9.3 ± 0.6	8.7 ± 1.2	8.4 ± 1.5	4.0 ± 1.7
Kinesiology (n=20)	100.0% (20/20)	8.6 ± 1.4	8.4 ± 1.5	8.6 ± 0.7	8.5 ± 1.3	8.0 ± 1.4	3.7 ± 1.5

**Table 2:** Scores for each of the evaluation criteria in practical questions Three evaluators assigned scores using a 10-point Likert scale (mean ± standard deviation)

Topics	Accuracy of the answer	Clarity	Relevance	Suitability level	Quality of distractors	Adequacy of rationale	Similarity
Musculoskeletal disorders (n=10)	100.0% (10/10)	9.3 ± 0.6	9.3 ± 0.7	8.8 ± 1.2	8.5 ± 1.4	8.1 ± 1.1	2.5 ± 1.1
Central nervous system disorders (n=10)	100.0% (10/10)	9.0 ± 1.1	8.9 ± 1.4	8.4 ± 1.5	7.9 ± 1.5	8.2 ± 1.3	2.6 ± 0.9
Internal organ disorders (n=10)	100.0% (10/10)	8.8 ± 1.2	9.3 ± 0.8	7.9 ± 1.6	7.7 ± 1.8	7.9 ± 1.5	2.7 ± 1.1

The average scores for clarity, relevance, suitability level, quality of distractors, adequacy of rationale, and similarity in general questions evaluated across three topics (anatomy, physiology, and kinesiology) are presented in Table 1.

Clarity showed the highest score for anatomy (9.0 ± 1.2), followed by physiology (8.7 ± 1.4) and kinesiology (8.6 ± 1.4). Relevance was rated equally for anatomy and physiology (8.5 ± 1.2), while kinesiology scored slightly lower (8.4 ± 1.5).

The suitability level was highest in physiology (9.3 ± 0.6), followed by anatomy (8.7 ± 1.1) and kinesiology (8.6 ± 0.7). The quality of distractors was rated highest in physiology (8.7 ± 1.2), with anatomy (8.4 ± 1.5) and kinesiology (8.5 ± 1.3) receiving comparable scores. The adequacy of rationale showed the highest score in physiology (8.4 ± 1.5), followed by anatomy (8.3 ± 1.2) and kinesiology (8.0 ± 1.4). Similarity scores, indicating how closely the generated questions resembled existing questions, were 4.0 ± 1.8 for anatomy, 4.0 ± 1.7 for physiology, and 3.7 ± 1.5 for kinesiology, showing a relatively low degree of overlap with prior exam questions.

As presented in Table 2, the practical questions covering musculoskeletal disorders, central nervous system disorders, and internal organ disorders demonstrated consistently high scores across multiple evaluation criteria. Clarity was rated the highest for musculoskeletal disorders (9.3 ± 0.6), followed by central nervous system disorders (9.0 ± 1.1) and internal organ disorders (8.8 ± 1.2). Relevance was similarly high across all categories, with central nervous system disorders scoring the highest (9.8 ± 1.4), followed by musculoskeletal disorders (9.3 ± 0.7) and internal organ disorders (9.3 ± 0.8). The suitability level showed some variation, with musculoskeletal disorders receiving the highest score (8.8 ± 1.2), followed by internal organ disorders (7.9 ± 1.6) and central nervous system disorders (8.4 ± 1.5). The quality of distractors was highest for musculoskeletal disorders (8.5 ± 1.4), with central nervous system disorders (7.9 ± 1.5) and internal organ disorders (7.7 ± 1.8) scoring slightly lower. The adequacy of the rationale was rated highest for central nervous system disorders (8.2 ± 1.3), followed by musculoskeletal disorders (8.1 ± 1.1) and internal organ disorders (7.9 ± 1.5). Similarity scores, reflecting the extent to which generated questions resembled existing ones, were 2.7 ± 1.1 for internal organ disorders, 2.6 ± 0.9 for central nervous system disorders, and 2.5 ± 1.1 for musculoskeletal disorders, indicating a relatively low overlap with prior exam questions.

## Discussion

This study represents the first attempt to use LLMs to generate national examination questions in physical therapy in a language structurally distinct from English, namely Japanese. Our findings suggest the possibility of generating MCQs, along with their correct answers and explanations, with relative ease. Notably, the generated questions exhibited exceptionally high accuracy and adhered to established question formats. The results indicate that LLMs can be leveraged to produce high-quality examination items, even in languages with linguistic structures distinct from English. Moreover, rather than merely replicating questions from the training data, the model successfully generated questions with a relatively high degree of novelty. These findings are similar to those in previous studies on medical examination question development [5,16].

In contrast, Ayub et al. reported that when using ChatGPT to generate dermatology board-style questions, only 40% of the 40 questions were deemed suitable for study preparation [17]. Similarly, Cheung et al. identified variability in the quality of MCQs generated by ChatGPT for graduate medical examinations [9]. These differences may stem from variations in data sources, prompt design, and evaluation methodologies. Ayub et al. [17] generated MCQs based on selected continuing medical education articles, while Chang et al. [14] used standard medical textbooks as reference materials. In contrast, our study utilized past questions from the Japanese National Licensure Examination for Physical Therapists to ensure that the generated content aligns with domain-specific competencies. Additionally, while previous studies primarily relied on standardized prompts, this study implemented a structured GPT framework integrated with RAG, enabling the generation of more contextually relevant questions. Furthermore, the application of RAG [12] and refined prompt engineering has been reported to significantly enhance the performance of LLMs [13,14], consistent with the present study's findings.

A notable contribution of this study is its focus on Japanese, a language structurally distinct from English. Prior research has predominantly focused on English-language examinations, leaving a gap in the current understanding of the utility of LLMs in linguistic and cultural contexts that differ significantly from English. The successful generation of high-quality MCQs in Japanese demonstrates the adaptability of LLMs and suggests that similar approaches could benefit languages with distinct grammatical and structural characteristics. This is particularly relevant for developing countries and populations facing linguistic disadvantages, where access to high-quality educational resources is often limited [18]. By facilitating the efficient generation of MCQs in diverse linguistic environments, LLMs could reduce educational disparities and improve access to high-quality learning and assessment materials.

Moving forward, using RAG and innovation in prompt design will likely remain critical components of similar endeavors. Furthermore, GPTs such as those developed in the present study, which can be easily shared and utilized online, hold significant potential for broad application among physical therapists and across educational settings in rehabilitation-related professions. However, as highlighted in this study, while the clarity and relevance (applicability) of the question stems were relatively strong, areas such as suitability based on difficulty (appropriate difficulty levels), quality of distractors (plausibility of incorrect options), and adequacy of the rationale (quality of explanations) received lower evaluations. This suggests that although GPTs successfully mimicked the structure of exam questions, they may not have fully captured the nuanced difficulty levels or academic rigor required for the national licensure examination for physical therapists [9]. Addressing these challenges will, therefore, require the development of larger and more carefully curated training datasets and further refinement of prompt configurations to enhance the quality and appropriateness of the generated questions.

Additionally, the generated explanations raise the challenging questions of defining target users and recognizing the extent of the evidence to be provided. The primary target users of the present study are students preparing for the national licensure examination for physical therapists. However, given the high accuracy and reliability required for such exams, the generated questions and explanations may not meet the standards necessary for direct use by students in their current form. The abstract nature of the prompt configuration used in this study may have influenced the depth and contextual accuracy of the explanations. Consequently, at this time, the questions generated by LLMs should be regarded as drafts, requiring human review and refinement prior to practical application. As noted in previous studies [11], human intervention is indispensable to ensure the quality, reliability, and educational validity of the final output.

Overall, the findings of this study demonstrate the potential of LLMs to generate MCQs that resemble those found in the Japanese National Licensure Examination for Physical Therapists with a reasonable degree of accuracy. This suggests that LLM-generated MCQs could improve the quality of educational resources and reduce educators’ workload. Nevertheless, several limitations must be acknowledged.

First, this study relied on a Likert scale-based evaluation, inherently subjective and dependent on the reviewers’ judgments. While this approach allowed for a simplified assessment of question quality, it lacks the precision needed for a more objective evaluation of LLM-generated MCQs. Future research should establish more standardized and objective evaluation criteria to ensure the reliability and validity of the generated questions, utilizing statistical methods for their assessment. Additionally, these evaluation criteria should be used to compare the actual national licensure exam questions, human-created mock exam questions, and AI-generated questions.

Second, this study did not assess item discrimination or the ability of the MCQs to differentiate between high- and low-performing examinees. Additionally, the effectiveness of LLM-generated MCQs has yet to be tested in real educational settings, such as in preparatory courses or simulated exams. Conducting such pilot studies would provide deeper insights into the strengths and limitations of AI-generated questions in practical applications and allow for necessary refinements. Third, although this study incorporated two years' worth of national licensure examination questions as pretraining data to generate mock exam questions, whether this volume was sufficient for optimal performance currently remains unclear. A larger and more diverse training dataset could potentially enhance the accuracy, originality, and overall quality of the generated MCQs. This study focused exclusively on the Japanese National Licensure Examination for Physical Therapists, which may limit its generalizability to other languages or licensure exams. Additionally, the scope of the questions was restricted to specific subject areas, and all of the domains of the actual examination were not comprehensively covered. Moreover, since the evaluation was conducted only on representative topics, the accuracy of AI-generated MCQs may not have been sufficiently examined. These limitations indicate that future research should evaluate the applicability of LLM-generated MCQs across different languages and educational or cultural backgrounds in examination contexts and further expand the range of covered topics to ensure a more representative assessment. Additionally, exam difficulty may vary by year. The selection of training data may introduce biases, potentially affecting the results, making the evaluation of biases in training data an important subject for future research.

Furthermore, exam difficulty may vary across years, while the training data selection could introduce biases, potentially influencing results. As such, future studies should consider the influence of such confounding factors to refine methodological approaches and improve the reliability of LLM-generated questions. Addressing these issues by incorporating more diverse training datasets and systematic evaluation frameworks will enhance the robustness and applicability of AI-generated MCQs in educational and assessment settings. Finally, while the Japanese National Licensure Examination for Physical Therapists includes image-based questions, our analysis focused exclusively on text-based MCQs. Expanding future research to include image-based questions would thus allow for a more comprehensive assessment of LLM capabilities in examination settings.

Despite these limitations, this study highlights the potential of LLMs to enhance educational and assessment methods in physical therapy and rehabilitation. As LLM technology advances and larger datasets become available, improvements in question quality, diversity, and validity are expected, increasing their utility in professional education. However, LLMs have inherent limitations, such as hallucination (generating incorrect but plausible information) and biases in training data, which may impact output accuracy. Additionally, while LLMs exhibit strong general language capabilities, their domain-specific knowledge may be incomplete without careful data curation, particularly in physical therapy.

Beyond question generation, future research should explore the integration of AI-generated MCQs into educational workflows. These questions could support adaptive learning, formative assessments, and automated feedback, improving student-teacher interactions and test preparation. However, practical implementation studies are needed to assess whether LLM-generated MCQs can effectively supplement traditional assessment methods. Comparative studies with existing question-generation approaches in different languages would further clarify their applicability across diverse educational contexts.

A human-in-the-loop approach should be adopted to maximize the practical utility of LLMs in education, where educators review and refine AI-generated questions before implementation. This approach ensures that questions meet the required academic and clinical standards while benefiting from AI’s efficiency in content generation. Additionally, LLMs could assist in question bank development, personalized learning materials, and real-time feedback systems, enhancing the overall quality of education in physical therapy.

Given these limitations, careful application and rigorous oversight are essential when incorporating LLMs into real-world education. Human supervision remains crucial to ensure accuracy, reliability, and contextual appropriateness. Moving forward, advancements in model architecture, dataset refinement, and prompt engineering will be necessary to fully harness the potential of LLMs in educational settings.

## Conclusions

Although challenges such as item discrimination, validation in real-world settings, and image-based question formats remain, the present study's results suggest that LLMs can enhance educational practices rather than fundamentally transform them. Further, their role in question generation and assessment still requires further empirical validation before broader claims can be made. Continued advancements in LLM technology and additional research are crucial for addressing these limitations and maximizing their impact on education in physical therapy and related fields. Additionally, the limitations inherent to LLMs, such as hallucination issues, biases in training datasets, and challenges in domain-specific knowledge, highlight the importance of careful supervision and human review during real-world application. Expanding the datasets used, refining evaluation frameworks, and conducting comparative analyses with different languages and educational systems will be essential to establishing LLMs as reliable and impactful tools for education and assessment. By addressing these technical and methodological challenges, LLMs may evolve into a more robust and practical resource for educational applications across various domains.

## Appendices

Supplemental material 1: excerpt of learning data stored in "knowledge"

This structure of data comprises 340 questions stored in "knowledge." While the actual data were in Japanese, they were translated into English for the purpose of this document.

Topics: Stroke

Question:

A 74-year-old female patient with left hemiplegia. According to the Brunnstrom method, her upper limb is classified as Stage II and her lower limb as Stage III. The muscle tone on the affected side is low, and voluntary muscle contractions are minimal. Moderate assistance is required for standing within parallel bars, as maintaining knee extension in the left lower limb is challenging, leading to knee buckling under weight-bearing conditions. Which of the following represents the correct Assessment entry in a problem-oriented medical record (POMR)?

1. Muscle weakness in the left lower limb.

2. Perform muscle-strengthening exercises for the left lower limb.

3. Decreased muscle tone in the left lower limb.

4. Use a long leg brace for the left lower limb and perform standing exercises.

5. Reduced weight-bearing capacity due to decreased muscle tone in the left lower limb.

Correct Answer: 5

Explanation:

The problem-oriented medical record (POMR) is a documentation method that focuses on the problems faced by the patient, aligning with the concept of problem-oriented medical care. This approach organizes information based on each problem and categorizes it into SOAP (subjective, objective, assessment, plan) for documentation. The content is structured as follows:

S (subjective): subjective information

O (objective): objective information

A (assessment): evaluation or assessment

P (plan): planning

Explanation of the Options:

1. & 3. Incorrect: Statements such as "Muscle weakness in the left lower limb" and "Decreased muscle tone in the left lower limb" fall under objective (objective information).

2. & 4. Incorrect: Actions like "Perform muscle-strengthening exercises for the left lower limb" and "Use a long leg brace for the left lower limb and perform standing exercises" are part of the plan (planning).

5. Correct: "Reduced weight-bearing capacity due to decreased muscle tone in the left lower limb" is an Assessment because it provides a reasoned interpretation based on Objective information.

Supplemental material 2: prompts for GPTs

This content was entered in a prompt field. While the actual data were in Japanese, they were translated into English for the purpose of this document.

# Prompt for creating practice questions for the National Examination for Physical Therapists

## User context

- Designed for students enrolled in physical therapy programs.

## About "knowledge"

- Column 1 contains the **topic**. 

- Column 2 contains the **question**. 

- Column 3 contains the **correct answer**. 

- Column 4 contains the **explanation**.

## Question creation

1. When a user inputs a **topic**, generate a **multiple-choice question with five options**.

2. Questions should reference past questions stored in "knowledge."

- Use the user-provided topic to reference column 1 in "knowledge" and identify relevant questions.

3. Generate questions by modifying past questions following these steps:

- Avoid repeating identical questions by altering options and content.

- Create new questions while adhering to the format of past questions.

- Include either **type A** (single correct answer) or **type X2** (two correct answers).

4. Provide explanations for both the **correct answer** and the **incorrect options**.

## Explanation creation

- Refer to the **explanations** stored in "knowledge" to generate detailed explanations for all answer choices.

## Output format

- **Multiple-choice question with five options**.

- Explanations should be concise yet specific.

- Example:

- *Which cranial nerve nucleus is located in the pons?*

1. Accessory nerve

2. Trochlear nerve

3. Facial nerve

4. Glossopharyngeal nerve

5. Hypoglossal nerve

## Randomization

- To prevent frequent repetition of past questions, introduce variations in the questions and answer choices.
